# Canine distemper viral infection threatens the giant panda population in China

**DOI:** 10.18632/oncotarget.23042

**Published:** 2017-12-08

**Authors:** Yipeng Jin, Xinke Zhang, Yisheng Ma, Yanchao Qiao, Xiaobin Liu, Kaihui Zhao, Chenglin Zhang, Degui Lin, Xuelian Fu, Xinrong Xu, Yiwei Wang, Huanan Wang

**Affiliations:** ^1^ College of Veterinary Medicine, China Agricultural University, Beijing 100193, People’s Republic of China; ^2^ College of Veterinary Medicine, Northwest A&F University, Shaanxi 712100, People’s Republic of China; ^3^ College of Animal Science, Zhejiang University, Zhejiang 310058, People’s Republic of China; ^4^ Foping National Nature Reserve, Shaanxi 723400, People’s Republic of China; ^5^ Beijing Zoo, Beijing 100044, People’s Republic of China

**Keywords:** canine distemper virus, giant panda (*ailuropoda melanoleuca*), canine (mixed breed), foping national nature reserve

## Abstract

We evaluated exposure to canine distemper virus (CDV) in eight wild giant pandas (*Ailuropoda melanoleuca*) and 125 unvaccinated domestic dogs living in and around Foping National Nature Reserve (FNNR), China. Seventy-two percent of unvaccinated domestic dogs (mixed breed) had neutralizing antibodies for CDV due to exposure to the disease. The eight wild giant pandas were naïve to CDV and carried no positive antibody titer. RT-PCR assays for *hemagglutinin* (*H*) gene confirmed the presence of CDV in 31 clinically ill dogs from several areas near FNNR. Genomic sequence analysis showed that the 21 canine CDV were highly homologous to each other and belonged to the Asian-1 genotype. They showed high homology with the GP01 strain sequenced from a fatally infected giant panda, suggesting cross-species infection. Observational and GPS tracking data revealed home range overlap in pandas and dogs around FNNR. This study shows that CDV is endemic in domestic dogs near FNNR and that cross-species CDV infection threatens the wild giant panda population.

## INTRODUCTION

Canine distemper is a highly contagious, fatal disease that affects domestic dogs (*Canis lupus familiaris)* and other species of the *Canidae*, *Mustelidae*, *Procyonidae*, *Felidae*, and *Ursidae* families [1–3]. Cross-species infection of canine distemper virus (CDV) from dogs to lion (*Panther oleo*) [1, 4, 5], African wild dog (*Lycaonpictus*) [6], Amur tiger (*Pantheratigrisaltaica)* [7], rhesus monkey (*Macacamulatta*) [2] and other wildlife is well documented. The first report of CDV infection in giant pandas (*Ailuropoda melanoleuca*) was at the Chongqing Zoo, China in 1997 [8]. A 2010 study demonstrated that 4/67 pandas at Wolong Research Center had developed anti-CDV antibodies [9]. In 2014-2015, a CDV epidemic was reported in captive pandas at the Shaanxi Rare Wild Animal Rescue and Breeding Center (SWARBC), China [10, 11]. The Foping National Nature Reserve (FNNR) is located in the Qinling Mountains, 57.7 km from SWARBC and has the highest density of free-ranging giant pandas [12, 13].

As of 2009, China had more than 27 million registered pet dogs and a large number of stray and unregistered domestic dogs with low vaccination rates [14]. Over 3000 reports of CDV in dogs in the last 10 years indicate that CDV is endemic in China [15]. Shaanxi province is one of the affected areas (Supplementary Figure 1). Many villagers in and around FNNR have domestic dogs for guarding purposes. Village dogs roam freely to forage for food, thereby increasing their contact with free-ranging pandas. Supplementary Figure 2 shows photographs of dogs in the habitat of wild giant pandas. Although Supplementary Figure 2 doesn’t show the predation relationship between pandas and dogs, dogs invaded into pandas’ habitat which give the chances touching dogs, excrement, which increases the possibilities of diseases pass from dogs to pandas. In addition, there is more than 1300 km of roads that cross the wild panda habitat in the Qinling Mountains increasing the opportunity for CDV infections.

Domestic dogs are a proven source of CDV for wild animals [16, 17] and CDV is often fatal in pandas [8, 10, 11]. Therefore, in this study, we studied the degree of CDV exposure in dogs near FNNR; analyzed if wild giant pandas in the area had developed protective anti-CDV antibodies; made genetic analysis of CDV strains between infected dogs around FNNR and infected giant pandas in order to determine contact among them.

## RESULTS

### Prevalence of anti-CDV antibodies in dogs and wild pandas near FNNR

Ninety of 125 dogs from the FNNR area tested positive for CDV antibodies (Table 1). Generalized linear mixed model (GLMM) analysis showed that villages had no effect on CDV antibody levels as we observed a variance of only 1.5%. Adult dogs showed higher CDV antibody levels than in juveniles (p <0.01) and puppies (p <0.01), whereas sex had no effect on CDV antibody levels (p >0.05).

**Table 1:** Seroprevalence of CDV antibodies in village domestic dogs near FNNR

Villages	Coordinates	Dog population	Total positive/Total tested	Percent Positive	Mean Titer± SD^*^
**Yueba**	33.5440°N, 107.8262°W	27	21/27	77.8%	1.74±1.38
**Daguping**	33.5894°N, 107.7743°W	67	19/32	59.4%	1.37±1.64
**Sanguanmiao**	33.6453°N, 107.7941°W	18	13/18	72.2%	1.33±1.41
**Longtanzi**	33.5152°N, 107.8871°W	22	16/22	72.7%	1.91±1.69
**Donghekou- Liangfengya**	33.6892-33.6975°N, 107.8921-107.9344°W	25	11/16	68.8%	1.69±1.66
**Xiaonanping**	33.6041°N, 107.9267°W	12	9/10	90.0%	1.8±1.23

*SD = Standard deviation.

**Figure 1:**
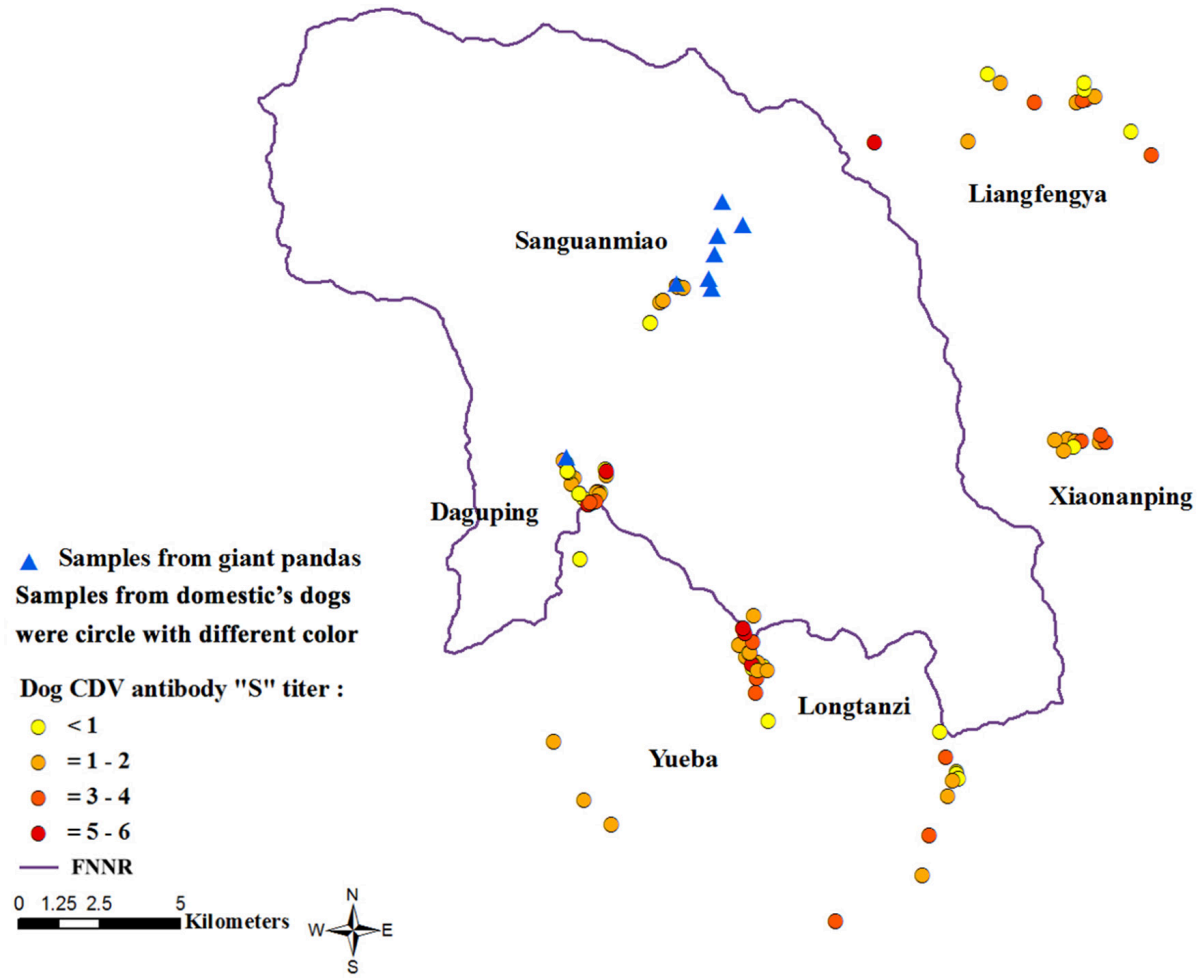
Anti-CDV antibody seroprevalence analysis The data shows anti-CDV antibody prevalence in 125 domestic dogs near FNNR. The map was constructed using ArcGIS10.0 software (http://www.esri.com/software/arcgis). Triangles indicate sampling sites for 8 wild giant pandas (all CDV negative). Circles indicate sampling sites of domestic dogs. Note: “S” means Scale of the titer; color scheme indicates anti-CDV antibody titers (negative to highest); Yellow dots = negative titer; Orange dots = S1-S2 titer; Red dots = S3-S4 titer; Dark red dots = S5-S6 titer.

As shown in Figure 1, the village with the highest antibody levels was Longtanzi (range S0-S5), whereas the village with the lowest antibody titers was Sanguanmiao (range S0-S6), which is located within FNNR in close proximity to giant pandas. The dogs were not vaccinated against CDV. The eight wild giant pandas tested negative for CDV on both Dot-ELISA (S0 titer) and neutralizing antibody assays (titer <1:4).

### RT-PCR and phylogenetic analysis

**Figure 2:**
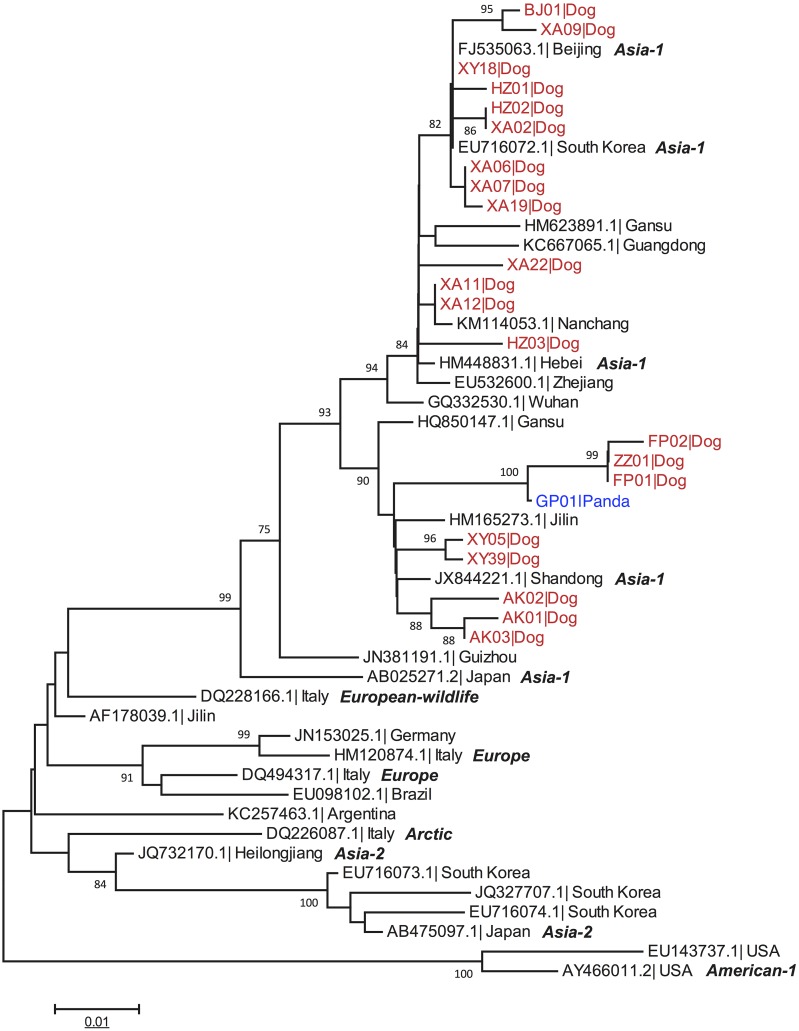
Phylogenetic analysis of domestic dog and giant panda CDV isolates The CDV genomes of 21 domestic dogs (in red) and 1 giant panda isolate (GP01, in blue) were compared with 28 CDV sequences downloaded from the GenBank. The phylogenetic tree was drawn to scale with MEGA6.0 software, with branch lengths measured in the number of substitutions per site. The tree with the highest log likelihood is shown. See Supplementary Table 2 for host type and keys to accession numbers.

RT-PCR analysis showed that 31 dogs from areas near FNNR were positive for CDV. The eight healthy wild giant pandas residing in FNNR were negative by RT-PCR. Figure 2 shows the maximum likelihood tree constructed using partial *H* gene of CDV strains isolated from 21 sick dogs, one infected giant panda and 28 full-length *H* gene sequences acquired from the GenBank. Then, the geographic distribution of various CDV genotypes was analyzed. The phylogenetic analysis showed the following: (1) all 21 canine strains clustered together in the Asian-1 genotype; (2) Xianyang (XY05, XY39) and Ankang (AK01, AK02, AK03) strains formed a sub-branch with Gansu, Shandong and Jilin strains; (3) Foping (FP01, FP02), Zhouzhi (ZZ01), Xianyang (XY18), Hanzhong (HZ01, HZ02, HZ03), Xi’an (XA02, XA06, XA07, XA09, XA11, XA12, XA19, XA22) and Baoji (BJ01) strains formed a sub-branch with strains from China, South Korea and Nanchang (Figure 2).

**Figure 3:**
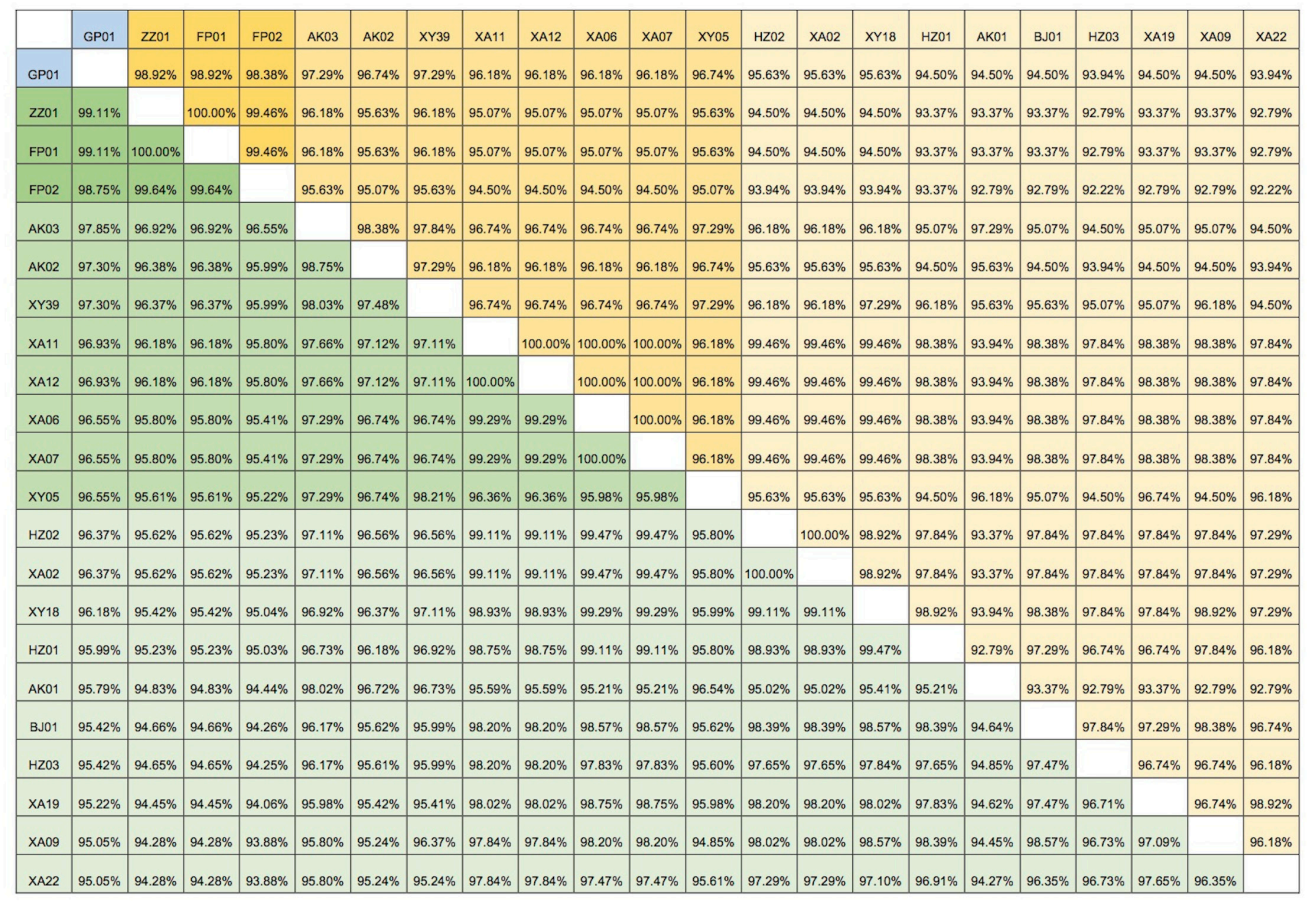
The Distance matrix analysis of CDV H gene sequences Pairwise percent identities of nucleotide and amino acid sequences between different strains of giant panda and domestic dogs CDV isolates were calculated using the PAUP software to generate a pairwise distance matrix. Giant panda strain is GP01 (in blue). The yellow boxes indicate amino acid similarity and green boxes indicate nucleotide sequence identity. Dark yellow/green boxes indicate high homology with the panda strain, while the light yellow/green boxes indicate low homology. See Supplementary Table 2 for the key to all strains.

As shown in Figure 3, the giant panda strain (GP01) showed high identity with the 21 wild-type strains (nt: 95.05%-99.11%, aa: 93.94%-98.92%). The ZZ01 and FP01 strains had the highest homology with GP01 (aa: 98.92%; nt: 99.11%). The XA22 and HZ03 strains showed least amino acid homology with GP01 (93.94%), whereas the XA09 and XA22 strains showed the least nucleotide homology with it (95.05%). The 21 wild-type dog strains shared 94.25-100.00% nucleotide sequence identity and 92.22 -100.00% amino acid similarity with each other. The nucleotide sequence of ZZ01 had the highest homology with FP01 (100.00%). FP02 strain had the lowest homology with HZ03 (94.25%). FP01 strain had the highest amino acid sequence homology with ZZ01 (100.00%), whereas the HZ03 strain had the lowest amino acid homology (92.22%) with FP02. Based on their level of homology with the panda strain GP01, there were 3 categories of the dog CDV strains. The ZZ01, FP01, and FP02 had the highest homology; AK03, AK02, XY39, XA11, XA12, XA06, XA07, XY05 strains had medium homology; and HZ02, XA02, XY18, HZ01, AK01, BJ01, HZ03, XA19, XA09, XA22 strains had the lowest homology.

### Temporal and spatial information analysis

**Figure 4:**
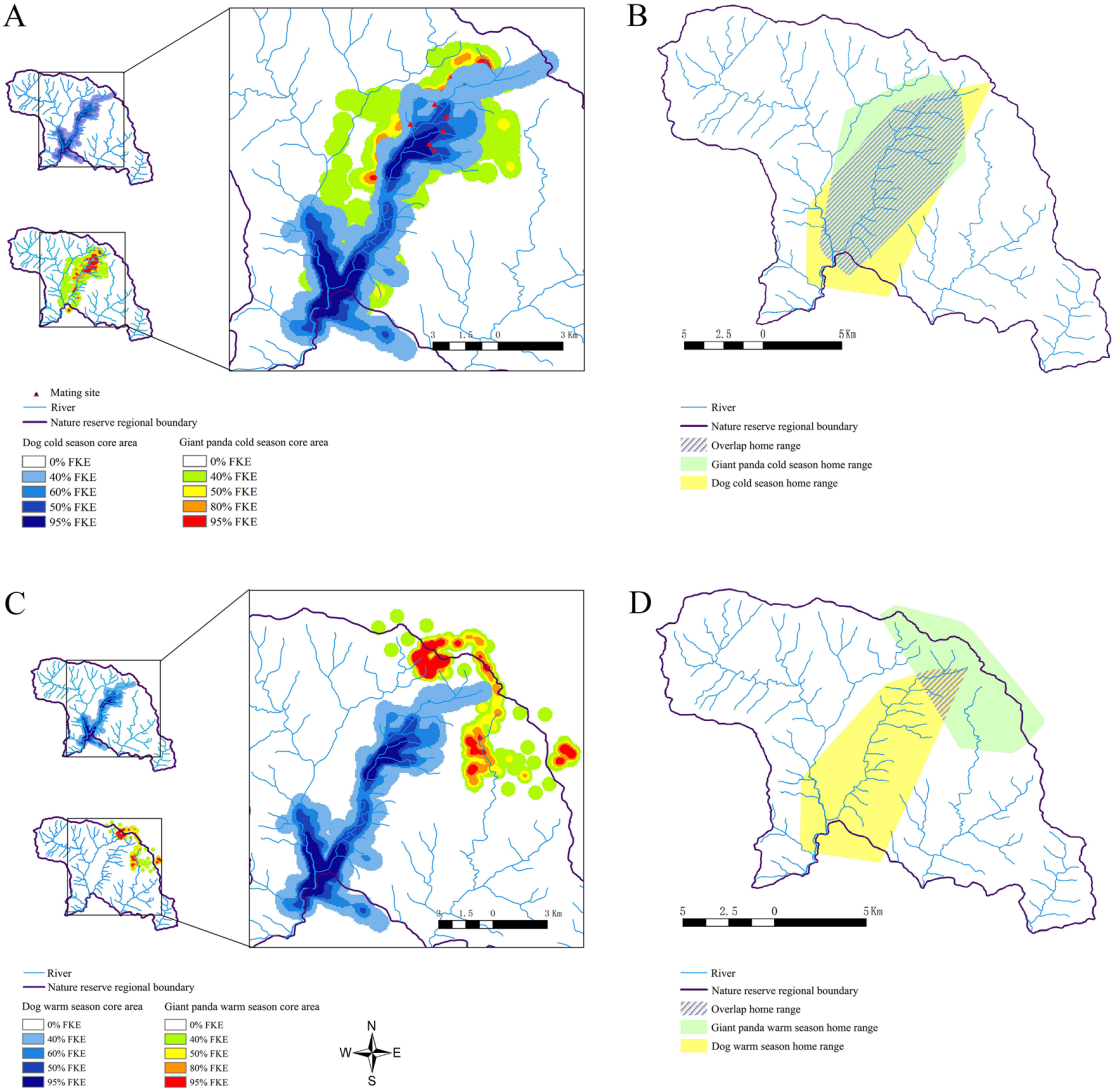
Home range analysis of domestic dogs and wild giant pandas Fixed kernel estimator (FKE) analysis of **(A)** winter and **(C)** summer core areas is shown for domestic dogs and wild pandas. Minimum convex polygon (MCP) analysis of **(B)** winter and **(D)** summer home ranges and overlap areas for domestic dogs and wild pandas is shown.

Based on giant panda migration patterns, the year is divided into a warm season (May - September) and cold season (October - April) [18]. The minimum convex polygon (MCP) home range analysis showed high overlap between domestic dogs and wild pandas during winter (47.8728 km^2^) and low overlap during summer (5.0037 km^2^). Fixed kernel estimator (FKE) showed that the core area overlap was 22.9729 km^2^ during winter and 0.0873 km^2^ during the summer. The home range changed minimally for domestic dogs during both seasons, but changed significantly for pandas (Figure 4). Pandas migrated 4km as the two seasons changed at a rate of 289 meters per day.

## DISCUSSION

Canine distemper is a common cause of morbidity and mortality in unvaccinated dogs [19]. It also affects common and endangered wild animal species worldwide [1–7, 17]. Domestic dogs and other related species serve as reservoirs of CDV and play an important role in disease transmission [6, 20, 21]. CDV epidemic occurs, which may be due to interaction between wildlife animals and domestic or feral dogs. A CDV outbreak resulted in extinction of the last remnant wild population of black-footed ferrets (*Mustelanigripes*) in 1985 [22]. CDV also causes recurrent mortality in African wild dogs (*Lyaconpictus*) [20]. Recent studies estimate that CDV infection may increase the 50-year extinction probability of Amur tigers by 6.3- 55.8% [7, 23]. The ability of CDV to infect multiple hosts combined with China’s large dog population with low vaccination rates raises concern for CDV epidemic in dogs affecting other domestic and wild animals like giant pandas [16, 24]. Because of low population density of giant pandas and their existence as small meta-popluations [25–27], the impact of CDV transmission from dogs to pandas cannot be ignored [28]. The co-existence of dogs in the habitat of pandas increases the possibility of disease transmission via the dog’s excreta.

The study of CDV in both captive and free-ranging pandas was necessary following the CDV outbreak in 2014-2015 at SWARBC [11]. Serological results in our study showed a high percentage of unvaccinated domestic dogs with anti-CDV antibodies suggesting exposure to CDV. This also demonstrated that CDV was endemic in regions around FNNR. Further, low antibody titers in dogs from Sanguanmiao village suggested that they were at risk of contracting CDV upon exposure. Since these dogs resided in prime giant panda habitat, they offered no safety buffer against the spread of CDV to the pandas.

None of the giant pandas tested in this study had positive antibody titer against CDV. Giant pandas are very susceptible to CDV, especially when they lack positive antibody titer [8, 11, 29]. Overall, mortality rates are very high for infected pandas [8, 11]. The giant pandas also suffer from poor nutrition, other health problems and exposure to extreme weather conditions in addition to high susceptibility to CDV infection.

Similar to previous reports, the *hemagglutinin*(H) gene sequences from CDV-infected dogs belonged to the wild-type Asia-1 cluster [30]. Genomic sequencing of CDV isolated from captive pandas also yielded Asia-1 type [10, 11]. Our study showed a high degree of homology between canine and giant panda CDV. The CDV strains isolated from dogs (FP01, FP02) near FNNR showed 99.11% homology with the strain isolated from a panda (GP01) that died of CDV at SWARBC [10]. Foping is only 15 kilometers away from FNNR. It also is a traffic hub of Shanxi and Sichuan including High Speed G5, national highway 108 and Xicheng high-speed rail with the national highway 108 connecting SWARBC and Foping directly. Our research showed four unique amino acid substitutions in H protein from the FP01, FP02, ZZ01 and GP01 strains, namely K281R, S300N, P340Q and Y549H. Y549H may determine the host barrier and suggests that the viral strain that caused mortality in the captive giant pandas in 2014-2015 was a mutant canine strain [11].

Temporal and spatial analysis showed that the home range overlap between free-ranging pandas and domestic dogs varied depending upon the season, primarily due to movement of the pandas. The home range of domestic dogs remained consistent throughout the year as they mainly foraged around villages and in the river valleys. However, the Qinling giant pandas migrated between seasons as they followed new growth of bamboo. When the pandas moved to higher elevations during summer [13, 31], there was minimal overlap between home range of pandas and dogs. However, during winters when the pandas gradually descended to lower elevations, there was significant overlap in home ranges between the two species (Figure 4). This interaction creates opportunities for potential CDV transmission from domestic dogs to pandas. Remote trail cameras have shown that both pandas and dogs used the same woodland trails within minutes of each other. The dog census in eight counties (Zhouzhi, Hu, Ningshaan, Foping, Yang, Chenggu, Liuba, Taibai) around FNNR in 2013-2014 ranged from 3.71 - 20.80 dogs/km^2^, which were sufficient to sustain spread of CDV [17].

During the mating season in March and April, the reproductively active pandas gather along the low-altitude hillsides where village dogs also reside. This also coincides with the mating season for domestic dogs. The intense roaming behavior increases direct and indirect inter-species contact that potentially could result in disease transmission. A previous review of 186 published reports of canine CDV cases based on temporal and spatial information revealed that highest number of CDV cases occurred from March to May [15, 32]. Therefore, the giant pandas are at greatest risk during their mating season. This is also critical since pathogenic CDV survives only for 48 hours at 25°C and for 14 days at 5°C [19].

CDV outbreak during the mating season can result in loss of fertility. In addition, close contact during mating would hasten transmission among animals and result in more deaths. Giant panda reproductive rates are very low as they reach sexual maturity at 5.5-6.5 years and give birth once every 2-3 years. Therefore, loss of fertility can dramatically affect the panda population. Out of the 1864 pandas in the wild [34], 345 reside in the Qinling Mountain region with around 80 residing in FFNR [33]. A substantial decrease in panda fertility could endanger their existence [25, 26].

Meta-analyses data shows that since 1995, CDV has affected many regions of China including giant panda reserves and breeding facilities (Supplementary Figure 1). Giant panda habitats in Shaanxi, Sichuan, and Gansu provinces are adjacent to three regions with high CDV prevalence (Regions I, II, IV). Any CDV outbreak within these three regions endangers giant panda populations. In addition, high trade, traffic, and human activity affect previously undisturbed habitats. As panda metapopulations become more fragmented due to human activity and loss of habitat, CDV virus poses a great threat to giant pandas in China [16, 20, 34–36]. Therefore, stringent disease prevention and management practices are necessary. Previous studies have focused on the risk of giant panda extinction due to environmental changes, food resources, genetics, and loss of habitat [12, 13, 31, 37]. Our study suggests that CDV infection has the potential to endanger the giant pandas

Most importantly, CDV is preventable. Vaccination strategies are needed to protect the giant pandas. Strict vaccination of domestic dogs with the commercial attenuated vaccine is needed [24, 38]. Canine vaccination buffer zones have combated CDV infection in lions and African wild dogs, and rabies in Ethiopian wolves [25, 39, 40]. The global threat posed by CDV to a number of endangered species has prompted mandatory vaccination of dogs [41]. Another prevention strategy is to vaccinate other known CDV reservoir species with recombinant vectored vaccines developed for those wild species. Regardless of the strategies employed, there is need for long-term monitoring and further studies.

Effective recombinant vaccines need to be developed for giant pandas. The multivalent, modified, live CDV vaccines for dogs have failed to produce positive antibody titer in giant pandas [42]. Apart from effective vaccines, safety of the pandas, practicality, cost, and unintended consequences of vaccination of in-target and non-target species need consideration [39, 43]. These pose great challenges and require long-term data collection from different species and collaboration between many research groups.

In conclusion, our study shows that cross-species infection of CDV from domestic dogs poses a serious threat to wild giant pandas in the FNNR region.

## MATERIALS AND METHODS

### Ethical statement

All animal study samples were collected according to animal protection laws of the People’s Republic of China. The Animal Care and Use Committee of China Agricultural University (approval number CAU-IACUC 2013-032) and the Foping National Nature Reserve approved this study.

### Study animals

Blood samples from 125 village dogs were collected to determine prevalence of anti-CDV antibody. The dogs were collared, photographed and surveyed on multiple occasions from 2013-2015. Data including sex, age, color, markings, ownership and vaccination history was also recorded.

Dogs were classified as puppies (0-4 months, n=12), juveniles (5-12 months, n=21) and adults (>12 months, n=92). Nasal, ocular or oral cavity swabs and blood samples were obtained from 31 clinically ill dogs from several areas near FNNR (Xi’an, Xianyang, Baoji, Ankang, Hanzhong, Zhouzhi County, Foping County; Supplementary Figure 3) for RT-PCR and genomic sequencing analysis between 2014-2015. Blood samples were obtained from superficial antebrachii under anaesthesia from 8 free-ranging giant pandas in FNNR (2 females, 6 males, average age 12 years) during the same period for antibody and RT-PCR assays. Please refer to the reference [44] about the specific method to obtain blood samples from giant pandas. The population density of giant pandas in the FNNR preserve is 80/300 km^2^.

### Sero-prevalence analysis

The ImmunoComb Canine VacciCheck IgG Antibody Dot-ELISA Test Kit (Biogal-Galed Laboratories, Kibbutz Galed, Israel) was used according to manufacturer’s instructions. Antibodies against CDV, canine infectious hepatitis and canine parvovirus were tested in blood from all 125 domestic dogs and eight giant pandas, immediately in the field. A score of 3-6 represents protective level of antibodies while scores 1 and 2 indicated low level of antibodies that were not enough to protect. The test did not discriminate between naturally acquired and vaccine-induced antibodies. Further, serum samples from giant pandas were analyzed for neutralizing CDV antibodies using a previously published methodology at the Central Laboratory of the Department of Clinical Veterinary Medicine, China Agricultural University [5]. Serum samples with antibody titers ≥1:32 (SN50) were considered positive.

### Virus detection and sequencing

Nasal, ocular or oral cavity swabs and blood samples were collected from 31 dogs with clinical signs of CDV and from 8 clinically healthy wild giant pandas. Total RNA was extracted from blood samples with EDTA using TRIzol reagent (TaKaRa, Dalian, China) according to the manufacturer’s instructions. The two pairs of primers (Supplementary Table 1; Beijing Sunbiotech Co. China) were used to sequence the 822 bp fragment of the *hemaglutinin* gene (*H*). Then, the partial *H* gene and the deduced amino acid sequences of isolates from domestic dog CDV were compared with H gene sequence of giant panda CDV [10] as well as 28 full-length H gene sequences at the GenBank (Supplementary Table 2). The deduced amino acid (aa) sequences were aligned using MEGA6.0 software (DNAStar Inc., Madison WI USA). Evolutionary history was inferred by the Maximum Composite Likelihood (MCL) method based on the Tamura-Neimodel [30, 45]. Neighbor-Join and BioNJ algorithms generated the initial trees from a matrix of pair wise distances estimated by the MCL method. The most superior log likelihood value was selected. Statistical significance of the phylogeny was estimated by bootstrap analysis over a 1000 replicates data set.

### Home range data collection

We tracked dogs from two villages (Sanguanmiao, n=18; Daguping, n=67) inside FNNR. Eleven dogs from Sanguanmiao traveled outside the village in three groups. Thirty dogs from Daguping traveled outside the village in five groups. One healthy, active dog from each village group (n=8) was fitted with a GPS collar (Lotek GPS3300SL, Lotek Wireless Inc., New market, Ontario Canada). Each dog’s position was recorded hourly from 06:00 to 18:00, with 12 position points taken every day. Recorded GPS data also included time, date, longitude, latitude, elevation, satellite number and the position precision of the attenuation factor. Based on previous studies [46, 47], GPS points (n=9429) were only selected if the 3D positioning and positional dilution of precision (PDOP) values were <10. The mobile loci of giant pandas in FNNR were obtained by observational methods (observing the appearance, footprints and feces of pandas) from 2012-2015. We obtained 2378 GPS sites with 378 sites from the Fourth Panda Survey^32^ and 2000 sites from field sightings.

Fixed kernel estimation (FKE) method estimated the core area of home ranges for both pandas and domestic dogs. The minimum convex polygon (MCP) method detected the overall overlap area between the home ranges of giant pandas and domestic dogs. The geographic information system software ArcGIS 10.0 (ArcGIS, ESRI, Redlands, CA USA) was used to generate the maps. The optimal bandwidth selected for the dogs was the equivalent line to 95% of the minimum value of the complete home domain.

### Statistical analysis

Generalized Linear Mixed Model (GLMM) fit analysis of CDV antibodies was estimated by maximum likelihood using the R language package. Sex and age were used as fixed factors while villages were considered as random effects.

## SUPPLEMENTARY MATERIALS FIGURES AND TABLES


