# Supplementary material for: Canine distemper viral infection threatens the giant panda population in China
Source: Oncotarget. 2017 Dec 8;8(69):113910–9. doi: 10.18632/oncotarget.23042 (PMC5768373; doi:10.18632/oncotarget.23042)
Supplement: Supplementary file 1 [file oncotarget-08-113910-s001.pdf]

Canine distemper viral infection threatens the giant panda population in China

SUPPLEMENTARY MATERIALS

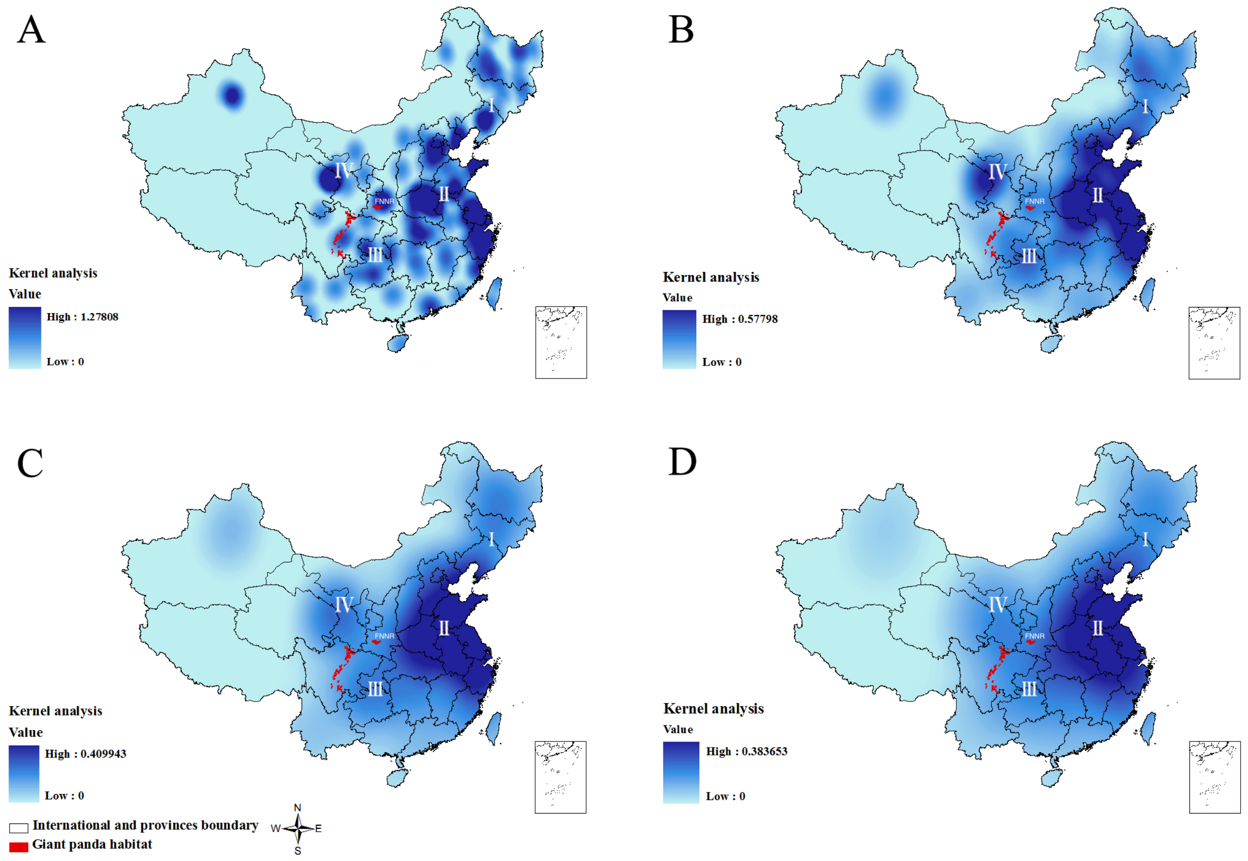

Supplementary Figure 1: Temporal and spatial analysis of CDV case reports from January 1995 to March 2015. Geographic information system software ArcGIS 10.0 (http://www.esri.com/software/arcgis); ESRI, Redlands, CA USA) was used to analyze spatial data. The data were simulated by kernel analysis and FKE band widths were set to 0.2 **(A)**, 0.4 **(B)**, 0.6 **(C)**, and 0.8 **(D)**, respectively. Kernel density analysis shows four regions of high CDV prevalence in Greater China. Region I in the northeast includes Heilongjiang, Jilin, and Liaoning provinces. Region II in the east includes the cities of Beijing, Tianjin and Shanghai, and the provinces of Hebei, Shandong, Henan, Jiangsu, Anhui, Zhejiang, and Hubei. Region III in the southwest includes Chongqing city, Sichuan, Yunan, Guizhou, and Guangxi provinces. Region IV in the northwest includes Gansu, Qinghai, Ningxia, and Shaanxi provinces. Simulation analysis also shows that CDV migration from east to west, and from south to north.

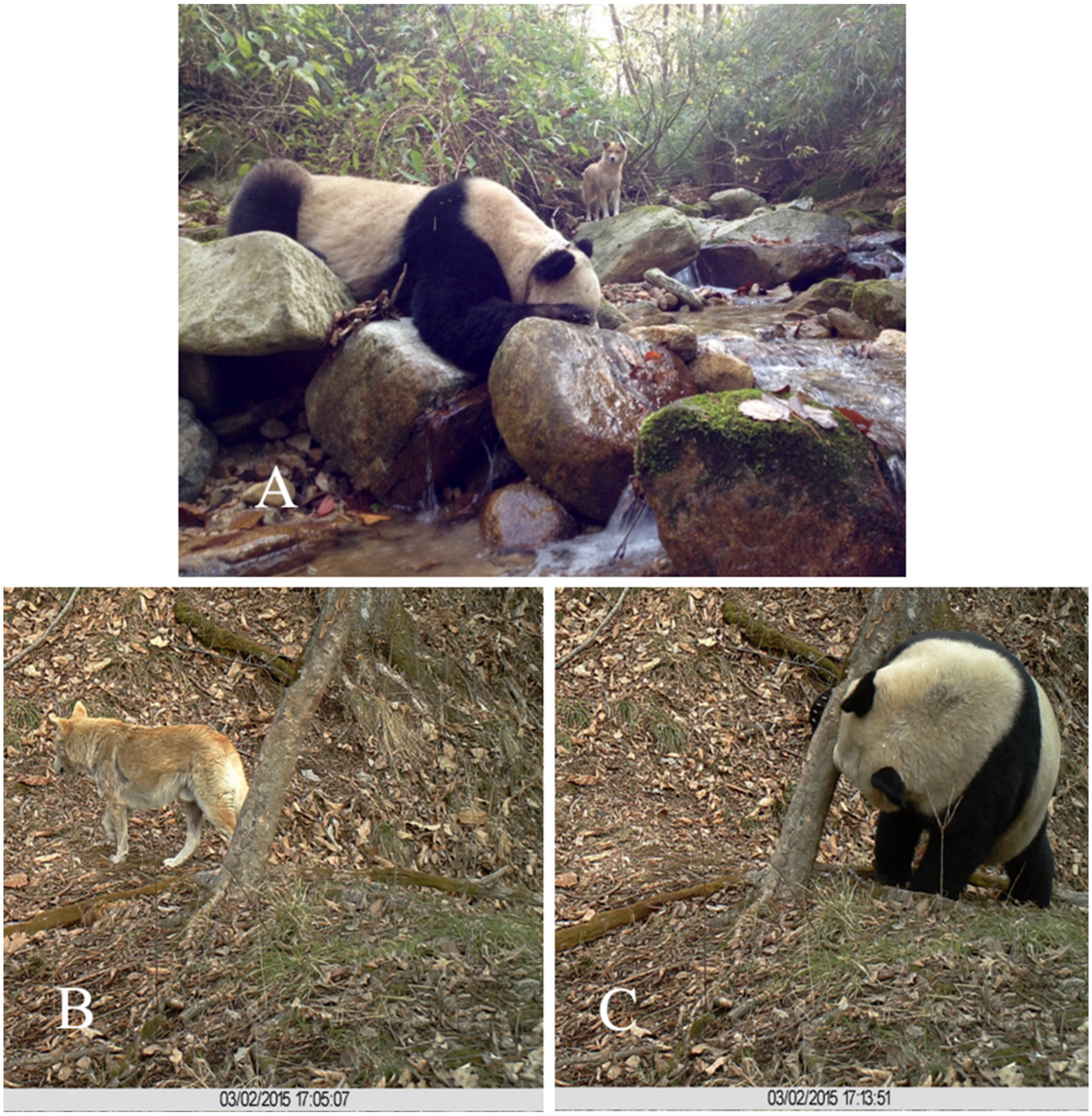

Supplementary Figure 2: Domestic dogs in wild giant panda habitats. **(A)** The photograph shows a 12-year old male panda drinking water that may be contaminated by excreta of the dog. The picture was shot in October, 2015, Paifang valley, FNNR. **(B & C)** The photographs show a male 3-year old dog followed by the panda sniffing the same place after 5 minutes.

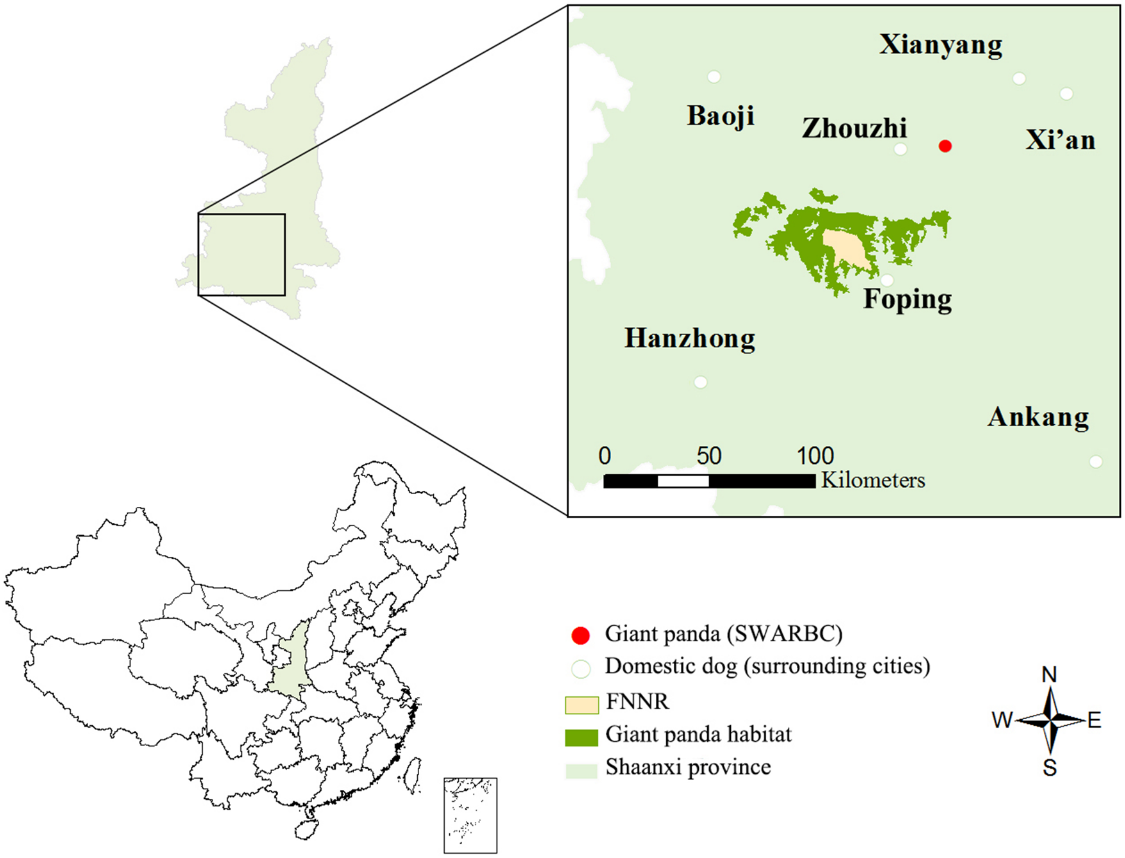

Supplementary Figure 3: Map of the study area. Map of Shaanxi province is shown in light green and the Qingling Mountain panda habitat is shown in dark green (zoomed). The red dot indicates the location of the giant panda breeding center (SWARBC) where a canine distemper virus outbreak occurred in 2014-2015[10,11]. White dots show locations where samples from 31 domestic dogs with CDV were obtained. Foping National Nature Reserve (FNNR) is shown in beige and is the site where samples from 8 wild, healthy giant pandas were obtained. The maps were drawn with ArcGIS 10.0 software (http://www.esri.com/software/arcgis).

Supplementary Table 1: CDV PCR primer pairs

PrimerPair	Sequence 5’- 3’	Position on Onderstepoort Strain Genome	Amplicon Size (bp)
**CDV-H-1**	5’-TGAGATTGGGTTACGGTTGC-3'	7381-7400	1470
	5’-CTGGTTGTAGAGTTGGCGAT-3’	8850-8831	
**CDV-H-2**	5’-AAGTTTTCCCCCTATCAGTCTC-3’	7668-7689	822
	5’-GGTCTCCTCTACCTGCTTTGTT-3’	8489-8468	

Supplementary Table 2: Nucleotide sequence accession numbers of CDV strains used in this study

Accession	Location	Subgroup	Host	Strain
AB025271	Japan	Asia-1	-	KDK-1
AB475097	Japan	Asia-2	-	M25CR
AF178039	China: Jilin	-	lesser panda	-
AY466011	USA	American-1	raccoon	98-2654
DQ226087	Italy	Arctic	dog	179/94
DQ228166	Italy	European-wildlife	dog	207/00
DQ494317	Italy	Europe	dog	324/03
EU098102	Brazil	-	dog	CDVBR1
EU143737	USA	-	-	Onderstepoort
EU532600	China, Zhejiang	-	dog	HZ026
EU716072	South Korea	Asia-1	dog	07Q72
EU716073	South Korea	-	dog	97Jindo
EU716074	South Korea	-	marten	98Marten
FJ535063	China, Beijing	Asia-1	alaskan malamute	_
GQ332530	China, Wuhan	-	dog	14
HM120874	Italy	Europe	red fox	SNP350/09/ITA
HM165273	China, Jilin	-	racoon dog	PS
HM448831	China, Hebei	Asia-1	fox	HeB(09)2
HM623891	China, Gansu	-	vero cells	GS0904-7
HQ850147	China, Gansu	-	vero cells	GS0812-4
JN153025	Germany	-	red fox	458/07
JN381191	China, Guizhou	-	dog	GZ2
JQ327707	South Korea	-	raccoon dog	R10-JB-004
JQ732170	China, Heilongjiang	Asia-2	raccoon dog	ZH(05)
JX844221	China, Shandong	Asia-1	fox	SD(12)2
KC257463	Argentina	-	Canisfamiliaris	Argentina 25
KC667065	China, Guangdong	-	dog	GZ0803
KM114053	China, Nanchang	Asia -1	dog	JX2
GP01	China, SWARBC	-	giant panda	Louguantai01
XY05	China, Xianyang	-	dog	-
XY18	China, Xianyang	-	dog	-
XY39	China, Xianyang	-	dog	-
AK01	China, Ankang	-	dog	-
AK02	China, Ankang	-	dog	-
AK03	China, Ankang	-	dog	-
BJ01	China, Baoji	-	dog	-
HZ01	China, Hanzhong	-	dog	-

(Continued)

Accession	Location	Subgroup	Host	Strain
HZ02	China, Hanzhong	-	dog	-
HZ03	China, Hanzhong	-	dog	-
XA02	China, Xi'An	-	dog	-
XA06	China, Xi'An	-	dog	-
XA07	China, Xi'An	-	dog	-
XA09	China, Xi'An	-	dog	-
XA11	China, Xi'An	-	dog	-
XA12	China, Xi'An	-	dog	-
XA19	China, Xi'An	-	dog	-
XA22	China, Xi'An	-	dog	-
ZZ01	China, Zhouzhi County	-	dog	-
FP01	China, Foping County	-	dog	-
FP02	China, Foping County	-	dog	-
